# Factors underlying the neurofunctional domains of the Addictions Neuroclinical Assessment assessed by a standardized neurocognitive battery

**DOI:** 10.1038/s41398-024-02987-9

**Published:** 2024-07-02

**Authors:** Tommy Gunawan, Jeremy W. Luk, Melanie L. Schwandt, Laura E. Kwako, Tonette Vinson, Yvonne Horneffer, David T. George, George F. Koob, Vijay A. Ramchandani, Nancy Diazgranados, David Goldman

**Affiliations:** 1 Office of the Clinical Director, National Institute on Alcohol Abuse and Alcoholism, National Institutes of Health, Bethesda, MD USA; 2 Human Psychopharmacology Laboratory, National Institute on Alcohol Abuse and Alcoholism, National Institutes of Health, Bethesda, MD USA; 3 Division of Treatment and Recovery, National Institute on Alcohol Abuse and Alcoholism, National Institutes of Health, Bethesda, MD USA; 4 Office of the Director, National Institute on Alcohol Abuse and Alcoholism, National Institutes of Health, Bethesda, MD USA; 5 Laboratory of Neurogenetics, National Institute on Alcohol Abuse and Alcoholism, National Institutes of Health, Bethesda, MD USA

**Keywords:** Human behaviour, Addiction, Scientific community

## Abstract

The Addictions Neuroclinical Assessment (ANA) is a neurobiologically-informed framework designed to understand the etiology and heterogeneity of Alcohol Use Disorder (AUD). Previous studies validated the three neurofunctional domains of ANA: Incentive Salience (IS), Negative Emotionality (NE) and Executive Function (EF) using secondary data. The present cross-sectional observational study assessed these domains in an independent, prospective clinical sample. Adults across the drinking spectrum (*N* = 300) completed the ANA battery, a standardized collection of behavioral tasks and self-report assessments. Factor analyses were used to identify latent factors underlying each domain. Associations between identified domain factors were evaluated using structural equation models. Receiver operating characteristics analyses were used to determine factors with the strongest ability to classify individuals with problematic drinking and AUD. We found (1) two factors underlie the IS domain: alcohol motivation and alcohol insensitivity. (2) Three factors were identified for the NE domain: internalizing, externalizing, and psychological strength. (3) Five factors were found for the EF domain: inhibitory control, working memory, rumination, interoception, and impulsivity. (4) These ten factors showed varying degrees of cross-correlations, with alcohol motivation, internalizing, and impulsivity exhibiting the strongest correlations. (5) Alcohol motivation, alcohol insensitivity, and impulsivity showed the greatest ability in classifying individuals with problematic drinking and AUD. Thus, the present study identified unique factors underlying each ANA domain assessed using a standardized assessment battery. These results revealed additional dimensionality to the ANA domains, bringing together different constructs from the field into a single cohesive framework and advancing the field of addiction phenotyping. Future work will focus on identifying neurobiological correlates and identifying AUD subtypes based on these factors.

## Introduction

Alcohol use disorder (AUD) is a chronic relapsing disease in which individuals often experience a compulsion to seek and use alcohol, loss of control over consumption, and negative affective states following cessation, leading to sustained problematic use [1]. The Diagnostic and Statistical Manual for Mental Disorders 5th edition (DSM-5) specifies 11 criteria for AUD, with individuals meeting ≥2 within any one year qualifying for an AUD diagnosis [2]. While this nosology offers diagnostic standardization and clinical reliability, it fails to address the heterogeneity of this disorder [3, 4], wherein individuals diagnosed with AUD often exhibit different medical histories and clinical presentations and as can be perceived from the many combinations of criteria sufficient for the diagnosis. Accordingly, experts have attempted to identify subtypes of AUD based on various genetic, physiological, psychological, sociocultural, and/or comorbidity indicators [5–7]. Despite these efforts, the lack of consensus on relevant AUD subtypes has limited adoption in clinical practice, highlighting the continuing need for a clinically relevant framework to understand this heterogeneity.

Recent approaches to psychopathology have utilized multidimensional frameworks to capture neuroscience-based domains across the functional spectrum and that are transdiagnostic in significance [3, 4, 8–10]. Variation in these domains is used to characterize clinical phenotypes of various psychopathologies and better understand the heterogeneity within diagnoses. One such approach is the Addictions Neuroclinical Assessment (ANA), a neuroscience-based clinical framework to understand the etiology and heterogeneity of AUD [9]. Based on the stages of the cycle of addiction [1, 11], the framework comprises three neurofunctional domains: Incentive Salience (IS), encompassing processes involved in reward, motivational salience, and habit formation (binge-intoxication stage); Negative Emotionality (NE), capturing negative affective states due to withdrawal and long-term drug use (withdrawal-negative affect stage); and Executive Function (EF), comprising cognitive functions related to inhibitory control, decision making, and planning of future goals (preoccupation-anticipation stage) [9, 12].

Kwako et al. provided initial evidence for the validity of the ANA domains using secondary data from the National Institute on Alcohol Abuse and Alcoholism (NIAAA) Natural History Protocol [12]. Factor analyses of selected neuropsychological assessments, primarily consisting of self-report measures, revealed three correlated factors concordant with the three domains. Established risk factors of AUD, such as family history of problematic alcohol use and childhood trauma, were significant predictors of these factors. Moreover, these factors demonstrated strong ability to distinguish individuals with AUD from ones without [12]. Independent replication of these results by other groups provided further evidence for ANA’s construct validity [13–17], predictive validity [17], and structural invariance [13]. Additional studies have also elucidated the domains’ potential neurobiological correlates [18], and extended the framework to other substance use disorders (SUDs) [19]. Furthermore, the ANA domains themselves parallel the three primary domains of the Research Domain Criteria (RDoC), proposed by NIMH as a research domain framework for psychiatric disorders in general, thus emphasizing the transdiagnostic value of the measures and the factors derived [8]. Though promising, these studies on the ANA construct in AUD had several key limitations, including use of secondary data from existing protocols, inconsistency in study design, and the use of primarily self-report measures, typically selected post hoc. Such measures lacked precision in assessing the proposed constructs as they were retrospectively selected and were inadequate in elucidating the latent dimensionality of these domains. For example, while cognitive psychology research suggests that executive function is a multidimensional construct [20], existing studies that validated the ANA framework had only found evidence of a unidimensional EF domain.

To optimally inform clinical practice, constructs with specific relevance to the etiology of AUD should also be evaluated for inclusion within the ANA framework. For example, sensitivity to alcohol is a key factor in the development of AUD [21], but it was not part of the initial ANA conceptualization. Empirical evaluation of these AUD-specific constructs in subsequent iterations of the ANA framework is necessary to better understand the etiology and heterogeneity of AUD. Furthermore, the comprehensive battery of assessments originally designed to assess the ANA domains was estimated to take 10 h to complete [9], representing a practical challenge for implementation whether in research or clinical practice.

To address these limitations, we developed a battery of neurocognitive behavioral tasks and questionnaires for the noninvasive assessment of the three ANA domains [9]. The goal of the current investigation is to (1) test the feasibility of the ANA battery, (2) characterize the underlying subfactors of the three neurofunctional domains and (3) identify associations between neurocognitive domain factors and AUD status.

## Methods

### Sample

Participants (*N* = 300) were enrolled between June 2018 and July 2021. Participants consisted of individuals admitted to a 28-day inpatient treatment program for AUD at the National Institutes of Health (NIH) Clinical Center (*n* = 181), and non-treatment-seeking individuals from the community recruited via advertisements and word of mouth who expressed interest in participating in alcohol-related research studies (*n* = 119). Eligibility criteria were completion of the NIAAA Natural History Protocol (NCT02231840), ≥18 years old, not under legal confinement, and not pregnant or breastfeeding. Twenty-nine participants whose data were included in the initial validation of the ANA domains [12] also completed the present study. Study sessions were conducted in the NIH Clinical Center in Bethesda, MD. All participants provided written consent before any study procedures. The protocol is registered in clinicaltrials.gov (NCT04946851) and was approved by the NIH institutional review board. Participants were compensated $100.

### Assessments

#### Addictions Neuroclinical Assessment (ANA) battery

The ANA battery consisted of a combination of neurocognitive behavioral tasks, self-report questionnaires, and clinical measures. Based on our previously proposed list of instruments [9], we piloted and selected each measure for inclusion based on: psychometric properties to minimize measurement bias (must have previously been shown to be valid and reliable), availability (available on the Millisecond Test Library [https://www.millisecond.com/download/library/]), feasibility (can be administered on the computer with a mouse and keyboard), and participation burden (each testing block, described below, should take no more than one h to complete). Due to these considerations, we did not include all of measures that were originally proposed. The list of instruments employed in the current battery and their psychometric properties can be found in Table 1.

**Table 1:** List of measures and their descriptives.

Domain	Assessment	Type	Construct	Variables/subscales	Reliability^a^	Assessment block^b^	Ref.
Incentive Salience	Alcohol Approach Avoidant Task (AAT)	Task	Approach bias for alcohol	AAT index for alcohol stimuli	0.99	Block 4	[56]
	Drinking Identity Implicit Association Task (IAT)	Task	Alcohol—self implicit association	D score	0.86	Block 2	[45]
	Alcohol Purchase Task (APT)^c^	Questionnaire	Alcohol Demand	Demand intensity	N/A	Block 3	[57]
				Demand elasticity (α)			
	Self-Rating of the Effects of Alcohol Questionnaire (SRE)	Questionnaire (Supplementary)	Alcohol Sensitivity	First Five	0.92	N/A	[58]
				Heaviest	0.94		
	Obsessive-Compulsive Drinking Scale (OCDS)	Questionnaire (Supplementary)	Compulsion for alcohol	Items #1, #11, #13^d^	0.90	N/A	[59]
	Alcohol Dependence Scale (ADS)	Questionnaire (Supplementary)	Alcohol-related thoughts	Item #18^e^	N/A	N/A	[60]
	Penn Alcohol Craving Scale (PACS)	Questionnaire (Supplementary)	Alcohol Craving	Total score	0.93	N/A	[61]
Negative Emotionality	Paced Visual Serial Addition Task (PVSAT-C)	Task	Distress Tolerance	Total correct	0.99	Block 4	[62]
				Quit during level 3			
	Cyberball	Task	Ostracism	Post-task emotional ratings^e^	0.90	Block 1	[63]
	Effort Expenditure for Rewards Task (EEfRT)	Task	Reward motivation	Proportion of hard-task choices	0.99	Block 2	[64]
	Snaith-Hamilton Pleasure Scale (SHAPS)	Questionnaire	Anhedonia	Total score	0.77	Block 1	[65]
	Connor-Davidson Resilience Scale (CD-RISC-25)	Questionnaire	Resilience	Total score	0.92	Block 3	[66]
	Experiences in Close Relationship Scale (ECR-R)	Questionnaire	Maladaptive attachment	Attachment-related anxiety	0.91	Block 3	[67]
				Attachment related avoidance	0.83		
	Positive and Negative Affect Scale (PANAS)	Questionnaire	Affect	Positive Affect	0.93	Block 4	[68]
				Negative Affect	0.89		
	Toronto Alexithymia Scale (TAS-20)	Questionnaire	Alexithymia	Total score	0.76	Block 4	[69]
	Inventory of Socially Supportive Behaviors (ISSB)	Questionnaire	Social Support	Total score	0.95	Block 4	[70]
	Montgomery-Åsberg Depression Rating Scale (MADRS)	Questionnaire (Supplementary)	Depression symptoms	Total score	0.93	N/A	[71]
	State-Trait Anxiety Inventory (STAI)	Questionnaire (Supplementary)	Trait anxiety	Trait anxiety score	0.96	N/A	[72]
	Buss-Perry Aggression Questionnaire (BPAQ)	Questionnaire (Supplementary)	Aggression	Physical Aggression	0.93	N/A	[73]
				Verbal Aggression			
				Anger			
				Hostility			
	NEO Personality Inventory – Revised (NEO-PI-R)	Ancillary	Personality	Extraversion score	0.87	N/A	[74]
				Agreeableness score	0.81		
				Neuroticism score	0.92		
Executive Function	Stop Signal Reaction Task (SSRT)	Task	Response Inhibition	Probability of responding in Stop trials	0.81	Block 4	[75]
				Covert stop signal reaction time	0.79		
	Visual Digit Span – Backwards	Task	Working memory	Two-error maximum length	0.79	Block 3	[76]
	Jumping to Conclusion (JTC) Beads Task	Task	Probability inference	Number of beads drawn	N/A	Block 4	[77]
	Trail Making Test (TMT)	Task	Task switching	Combined total time	0.99	Block 3	[78]
	Manikin Test of Spatial Orientation (Manikin)	Task	Spatial ability	Proportion correct	0.97	Block 2	[79]
	Continuous Performance Task (CPT)	Task	Attention	D-prime	0.94	Block 1	[80]
	Metacognition Questionnaire (MCQ-30)	Questionnaire	Metacognition	Lack of cognitive confidence	0.88	Block 2	[81]
				Positive beliefs about worry	0.86		
				Cognitive self-consciousness	0.78		
				Negative beliefs about uncontrollability and danger	0.86		
				Need to control thoughts	0.72		
	Multidimensional Assessment of Interoceptive Awareness (MAIA)	Questionnaire	Interoceptive Awareness	Noticing	0.78	Block 1	[82]
				Not distracting	0.63		
				Not worrying	0.53		
				Attention regulation	0.89		
				Emotional awareness	0.83		
				Self-regulation	0.85		
				Body listening	0.84		
				Trusting	0.89		
	Barratt Impulsivity Scale (BIS)	Questionnaire (Supplementary)	Impulsivity	Attentional impulsiveness	0.81	N/A	[83]
				Motor impulsiveness	0.63		
				Nonplanning impulsiveness	0.81		
	UPPS-P Impulsivity Scale (UPPS-P)	Questionnaire (Supplementary)	Impulsivity	Negative Urgency	0.94	N/A	[84]
				Lack of premeditation	0.85		
				Lack of perseverance	0.84		
				Sensation Seeking	0.83		
				Positive Urgency	0.92		
	Monetary Delay Discounting Task	Questionnaire (Supplementary)	Delay Discounting	Discounting rate *k*	N/A	N/A	[85]

List of measures used in the Addictions Neuroclinical Assessment battery and their psychometric properties. Supplementary questionnaires were collected as part of a separate protocol. See text for details.

^a^Internal consistency (Cronbach’s *α*) was reported for questionnaires and split-half reliability (Pearson’s *r*) was reported for behavioral tasks.

^b^Assessment battery was split into four testing blocks. Blocks were presented in random order. See text for details.

^c^Breakpoint, O_max_, and P_max_ could not be computed due to insufficient price range.

^d^OCDS item #1: How much of your time when you’re not drinking is occupied by ideas, thoughts, impulses or images related to drinking?

OCDS item #11: If you were prevented from drinking alcohol when you desired a drink, how anxious or upset would you become?

OCDS item #13: How strong is the drive to consume alcohol beverages?

^e^ADS item #1: Do you almost constantly think about drinking alcohol?

#### Supplementary measures

Additional self-report and clinical measures relevant to the ANA domains but collected separately under the Natural History Protocol were included in the present analyses (Table 1). These measures, including several scales and individual items that were part of the initial validation study [12], provide additional depth and breadth to the assessment of the three domains by capturing information not otherwise included in the ANA battery. We specifically included individual items that had previously been used to validate the ANA domains (OCDS #1, #11, #13, and ADS #18) to ensure consistency and replicability of previous findings [12, 14, 15].

#### Demographic characteristics and AUD status

Self-reported age, sex, race, ethnicity, education, and annual household income were assessed. Past year and lifetime DSM-5 AUD and other DSM diagnoses were determined using the Structured Clinical Interview for DSM-5 (SCID-5) [22].

#### Alcohol-related variables

The Alcohol Use Disorders Identification Test (AUDIT) was used to evaluate problematic drinking [23]. Family history of alcohol problems were quantified using the Family History Density (FHD) score [24]. Past 90-day drinking was evaluated using the Timeline Followback [25].

### Procedures

Informed consent was obtained prior to any study procedures. All participants had a negative breath alcohol concentration prior to the start of the battery. Participants that were in the inpatient treatment program were tested after completion of detoxification and documentation of no withdrawal symptoms (using the Clinician Institute Withdrawal Assessment, CIWA-Ar [26]). The ANA battery was administered in four testing blocks (Table 1), with the order of the blocks randomized across participants. Order of assessments within each block was not randomized. Within each block, behavioral assessments always preceded questionnaires. Participants were given the opportunity to take a 15 min break between blocks to reduce potential response bias due to fatigue. All behavioral tasks were administered using Inquisit 5 (Millisecond Software LLC, Seattle, WA) and are available online (https://www.millisecond.com/download/library/).

### Statistical analyses

#### Descriptives

Descriptive statistics of demographic and alcohol-related measures were computed as means, standard deviations, and correlations. Means, medians, standard deviations, skewness, kurtosis, missingness, and psychometric properties of the ANA assessments (split-half reliabilities for behavioral tasks, Cronbach’s *α*s for questionnaires) were also computed.

#### Factor analyses

The dataset was randomly split into a testing (*n* = 150) and validation set (*n* = 150). Factor analyses were conducted separately by domain. For each domain, exploratory factor analyses (EFAs) were conducted on the testing set to identify latent factors using robust weighted least squares estimator with geomin rotation and full information maximum likelihood. The number of factors extracted were determined using fit indices and factor interpretability. A root mean square error of approximation (RMSEA) value of ≤0.06 indicated good model fit, and a comparative fit index (CFI) and Tucker-Lewis index (TLI) value of ≥0.95 indicated optimal model fit (≥0.90 indicated acceptable fit [27]). Indicators with loadings of ≥0.35 were retained. If a satisfactory solution was not produced, the indicator with the lowest communality was dropped and the EFA was re-ran until a satisfactory solution was reached. Confirmatory factor analyses (CFAs) for each domain were conducted using the validation set based on solutions from the EFAs. Model modifications were evaluated and applied based on modification indices (MIs > 10.0) and theoretical interpretability.

#### Structural equation models

Structural equation modeling (SEM) was subsequently used to combine all three domain structures into a single model using the full sample. Additional cross-domain paths were considered based on modification indices (MIs > 10.0) and theoretical interpretability. Partial correlations, controlling for sex, age, and race, were estimated to identify the strength of associations across the domain factors. To test for model robustness, leave-one-out analyses were conducted to ensure that no single indicator was disproportionately driving the domain factors. Missing data were imputed using full information maximum likelihood.

#### Differences in factor scores by AUD status and problematic drinking

Standardized factor scores adjusted for age, sex, and race were extracted from the final model and compared between individuals with and without a current AUD diagnosis using Student’s *t*-test. Receiver operating characteristic (ROC) area under the curve (AUC) analysis was also conducted to test the ability of each domain factor to predict problematic drinking (total AUDIT score >8) and current AUD status.

Factor analyses and structural equation models were conducted in Mplus 8.4 [28]. All other analyses were conducted in R version 4.2.2 [29]. A cutoff of *p* < 0.05 was used to indicate statistical significance. The Benjamini-Hochberg procedure was used to adjust for multiple comparisons.

## Results

### Demographic and clinical characteristics

The sample was 41.0% female, with an average age of 42.5 years. Approximately half (50.5%) of the sample identified as White Caucasian, 36.0% as African American, 3.3% as American Indian or Alaskan Native, 0.7% as Asian, and 9.4% as Other. Participants were on average 42.5 years old (SD = 13.1), with 14.3 years of education (SD = 3.0). Approximately one-third of the sample (32.3%) had an annual household income of <$20,000, 44.1% had $20,000–$75,000, and 23.6% had >$75,000. There were 209 (69.7%) individuals who met DSM-5 criteria for current (past year) AUD, with 90.1% qualifying as having severe AUD (≥6 or more criteria). Of these, 175 (85.8%) of them were seeking treatment for their AUD at the NIH Clinical Center. Compared to individuals without AUD, individuals with AUD were more likely to be male, were older, and reported heavier and more frequent alcohol consumption, greater AUDIT scores, and higher FHD scores (*p*’s < 0.001; Table 2). Additional information about the sample including psychiatric comorbidity and socioeconomic status are shown in Table 2.

**Table 2:** Demographic and alcohol use information.

	AUD (*n* = 210)	non-AUD (*n* = 89)	Full sample (*n* = 300)^a^	*p*^e^
	Counts (%)/Mean (SD)	Counts (%)/Mean (SD)	Counts (%)/Mean (SD)	
Sex				<0.001
Male	137 (65.2%)	39 (43.8%)	177 (59.0%)	
Female	73 (34.8%)	50 (56.2%)	123 (41.0%)	
Age (years)	44.5 (12.6)	38.3 (13.5)	42.5 (13.1)	<0.001
Race				0.10
Caucasian White	113 (53.8%)	38 (42.7%)	151 (50.5%)	
African American	67 (31.9%)	41 (46.1%)	108 (36.0%)	
American Indian/Alaskan Native	9 (4.3%)	1 (1.1%)	10 (3.3%)	
Asian	2 (1.0%)	0 (0.0%)	2 (0.7%)	
Other	19 (9.0%)	9 (10.1%)	27 (9.4%)	
Annual household income ($/year)^b^				<0.001
<$20,000	83 (39.9%)	13 (14.6%)	96 (32.3%)	
$20,000–$75,000	89 (42.8%)	42 (47.2%)	131 (44.1%)	
>$75,000	36 (17.3%)	34 (38.2%)	70 (23.6%)	
Hispanic	21 (10.3%)	7 (7.9%)	29 (9.7%)	0.78
Education (years)	13.8 (3.0)	15.8 (2.7)	14.3 (3.0)	<0.001
Treatment-seeking for AUD^c^	181 (86.2%)	0 (0.0%)	181 (60.3%)	<0.001
DSM-5 AUD severity^d^				<0.001
Mild (2–3 symptoms)	13 (6.2%)	0 (0.0%)	7 (2.3%)	
Moderate (4–5 symptoms)	12 (5.7%)	0 (0.0%)	12 (4.0%)	
Severe (6 or more symptoms)	183 (87.1%)	0 (0.0%)	173 (57.7%)	
No. of drinking days (past 90 days)	69.0 (24.8)	15.1 (16.1)	55.6 (32.1)	<0.001
Average drinks per drinking day (past 90 days)	14.7 (10.6)	2.0 (1.1)	11.3 (10.6)	<0.001
No. of heavy drinking days (past 90 days)	65.3 (36.2)	1.6 (4.0)	48.6 (41.7)	<0.001
AUDIT total score	26.0 (8.6)	2.9 (2.5)	20.1 (12.6)	<0.001
Family History Density	0.14 (0.16)	0.03 (0.07)	0.11 (0.15)	<0.001
Any DSM-5 depressive disorders	113 (53.8%)	9 (10.2%)	122 (40.9%)	<0.001
Any DSM-5 anxiety disorders	52 (24.8%)	1 (1.1%)	53 (17.8%)	<0.001
Any DSM-5 trauma disorders	80 (38.3%)	5 (5.7%)	85 (28.6%)	<0.001

Sociodemographic, clinical, and alcohol use characteristics of the sample.

*AUD* alcohol use disorder, *DSM-5* Diagnostic and Statistical Manual for Mental Disorders 5th edition, *AUDIT* Alcohol Use Disorder Identification Test.

^a^Current AUD status was missing for one individual.

^b^Three individuals (2 AUD and 1 non-AUD) had missing data on annual household income.

^c^Treatment-seeking individuals were individuals who were admitted to the alcohol inpatient clinic at the NIH Clinical Center.

^d^AUD severity was missing for two individuals due to technical issues.

^e^*p* values were based on independent samples Student’s *t* test for continuous variables and Chi-square test for categorical variables.

### ANA blocks and assessments

Descriptive statistics, correlation matrix, and missing data rates of the ANA battery assessments are found in Table S1. Average time to complete all four blocks was 139 ± 28 mins (median = 132 min, range = 91–346 min), excluding time taken for breaks and collection of supplementary measures from the Natural History Protocol. Of the 300 participants, 98% (*n* = 294) completed all four blocks. One individual completed three blocks (voluntarily discontinued), 4 completed two blocks (*n* = 2 voluntarily discontinued; *n* = 2 disqualified due to noncompliance), and 1 completed one block (disqualified due to comprehension issue). Missing data varied across measures (median missing data rate = 1.33%), with the Experiences in Close Relationship Scale having the highest missing data (26.0%) due to technical software issues.

### Factor structures

**Fig. 1:**
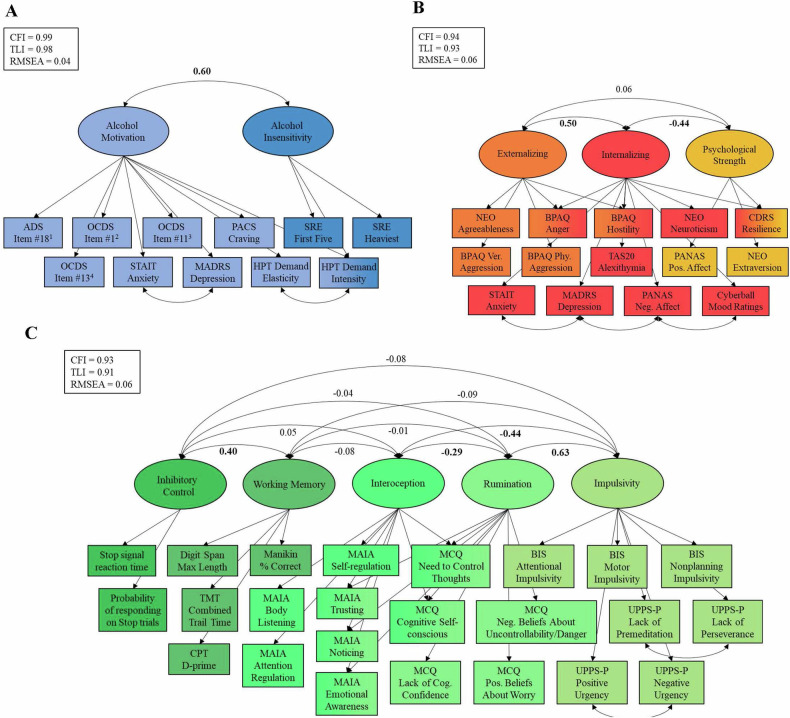
Multifactorial structure of the neurofunctional domains of the Addictions Neuroclinical Assessment. Latent structure of the (**A**) Incentive Salience, (**B**) Negative Emotionality, and (**C**) Executive Function domains. Values above double-headed arrows indicate correlation coefficients between factors. NEO Agreeableness loaded negatively to Externalizing. TMT Combined Trail Time was negatively loaded to Working Memory. ^1^ ADS #18 = Do you almost constantly think about drinking alcohol?. ^2^ OCDS #1 = How much of your time when you’re not drinking is occupied by ideas, thoughts, impulses, or images related to drinking?. ^3^ OCDS #11 = If you were prevented from drinking alcohol when you desired a drink, how anxious or upset would you become?. ^4^ OCDS #13 = How strong is the drive to consume alcoholic beverages?. ADS Alcohol Dependence Scale, OCDS Obsessive Compulsive Drinking Scale, STAIT State-Trait Anxiety Inventory, Trait version, MADRS Montgomery-Åsberg Depression Rating Scale, PACS Penn Alcohol Craving Scale, SRE Self-Reported Effects of Alcohol Questionnaire, HPT Hypothetical Purchase Task, BPAQ Buss-Perry Aggression Questionnaire, CDRS Connor-Davidson Resiliency Scale, TAS20 Toronto Alexithymia Scale, PANAS Positive and Negative Affect Scale, TMT Trail Making Test, CPT Continuous Performance Task, MAIA Multidimensional Assessment of Interoceptive Awareness, MCQ Metacognition Questionnaire, BIS Barratt Impulsivity Scale, UPPS-P UPPS-P Impulsive Behavior Scale.

For incentive salience, the two-factor and three-factor solutions from the EFA provided the best fit (Table S2). However, the three-factor solution included two factors with only one indicator that showed a loading of >0.35, thus the two-factor solution was used for subsequent analyses (CFI = 0.99, TLI = 0.98, RMSEA [90% CI] = 0.06 [0.04, 0.08]). The first factor, alcohol motivation, was indicated by variables related to alcohol obsessions and compulsions, craving, anxiety symptoms, depressive symptoms, demand intensity and elasticity. The second factor, alcohol insensitivity, was indicated by demand intensity and scores on the Self-Rating of the Effects of Alcohol Questionnaire (SRE). Initial CFA with the two-factor structure resulted in unsatisfactory model fit (CFI = 0.98, TLI = 0.98, RMSEA [90% CI] = 0.08 [0.06, 0.10]). We specified correlations between demand intensity and demand elasticity, and between Montgomery-Åsberg Depression Rating Scale (MADRS) and State-Trait Anxiety Inventory—Trait (STAIT) scores. A satisfactory model fit was achieved with both the validation (CFI = 0.98. TLI = 0.98, RMSEA = 0.06) and full sample (CFI = 0.99, TLI = 0.98, RMSEA [90% CI] = 0.04 [0.02, 0.06]; Fig. 1A).

For negative emotionality, a four-factor solution provided adequate fit to the data (CFI = 0.97, TLI = 0.94, RMSEA [90% CI] = 0.06 [0.03, 0.08]; Table S3). The first factor consisted of state negative affect (Positive and Negative Affect Scale (PANAS) negative affect score and post-Cyberball emotional ratings), the second factor consisted of internalizing emotional processes (e.g., neuroticism, depression, anxiety, stress), the third factor captured psychological strength (e.g., resilience, positive affect), and the fourth factor comprised measures of aggression. CFA of the four-factor solution produced poor model fit (CFI = 0.87, TLI = 0.83, RMSEA [90% CI] = 0.09 [0.08, 0.11]), thus we (1) combined the first and second factor due to their conceptual similarity, and (2) applied several modifications (specifying Buss-Perry Aggression Questionnaire—Anger subscale score as an indicator of Factor 1, allowing post-Cyberball emotion ratings to correlate with PANAS negative affect score, and MADRS depression scores to correlate with STAIT anxiety scores and PANAS negative affect score). These steps resulted in acceptable fit in both the validation set (CFI = 0.92, TLI = 0.90, RMSEA [90% CI] = 0.08 [0.06, 0.10]) and the full sample (CFI = 0.96, TLI = 0.94, RMSEA [90% CI] = 0.06 [0.05, 0.07]). This final model consisted of three factors: internalizing—capturing negative emotions directed inwards; externalizing—characterized by negative behaviors directed outwards; and psychological strength—the ability to cope with stress (Fig. 1B).

For executive function, the EFA did not converge to a solution for models with 4-factors or higher. We removed the indicator with the lowest communality to reduce the model complexity (Jumping To Conclusions Beads Task), after which a 5-factor solution provided the best fit (CFI = 0.94, TLI = 0.90, RMSEA [90% CI] = 0.06 [0.05, 0.07]; Table S2). Initial CFA model fit was poor (CFI = 0.83, TLI = 0.81, RMSEA [90% CI] = 0.09 [0.08, 0.10]), but was improved with several modifications (allowing UPPS-P positive urgency score to correlate with UPPS-P negative urgency score, UPPS-P lack of perseverance score to correlate with UPPS-P lack of premeditation score; allowing Multidimensional Assessment of Interoceptive Awareness (MAIA) noticing, emotional awareness, body listening, cognitive self-conscious, and need to control thoughts subscale scores to load across two factors): CFI = 0.91, TLI = 0.90, RMSEA [90% CI] = 0.06 [0.05, 0.08] in the validation set; CFI = 0.93, TLI = 0.91, RMSEA [90% CI] = 0.06 [0.05, 0.06] in the full sample). This final model with five factors included: inhibitory control—the inhibition of prepotent responses; working memory—the ability to hold and manipulate information in short-term memory; interoception—the ability to detect internal bodily states; rumination—worry and lack of control over thoughts; and impulsivity—the tendency to act without thinking (Fig. 1C).

### Full structural model

The three models from each domain were combined into a single model using the complete dataset (CFI = 0.88, TLI = 0.87, RMSEA [90% CI] = 0.06 [0.05, 0.06]). Leave-one-out analyses showed that factor scores did not significantly differ from the original model when each indicator was iteratively removed from the model (*r*’s = 0.90–1.00), suggesting that no single indicator unduly influenced the estimation of the domain factors. Full model parameters are found in Table S3. Partial correlations revealed varying degrees of associations between factors (|*r*|’s = 0.00 to 0.89; Table S4). The strongest correlations were between internalizing and impulsivity (*r* = 0.89), impulsivity and rumination (*r* = 0.87), and alcohol motivation and impulsivity (*r* = 0.81). The weakest correlations were found with inhibitory control (only moderately correlated with working memory, *r* = 0.45) and working memory (only weakly correlated with alcohol motivation, *r* = −0.14, alcohol insensitivity, *r* = −0.18, and externalizing, *r* = 0.16). Associations between factor scores and age, sex, race, and family history density are found in Table S5.

### Differences by AUD diagnosis and problematic drinking

**Fig. 2:**
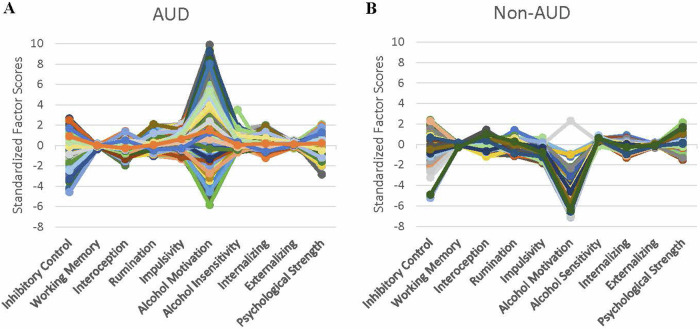
Heterogeneity of neurofunctional domain factors across the sample. Profile plots for each individual subject’s standardized factor scores for individuals (**A**) with AUD and (**B**) without AUD, adjusted for age, sex, and race. Each colored line indicates a unique individual.

**Fig. 3:**
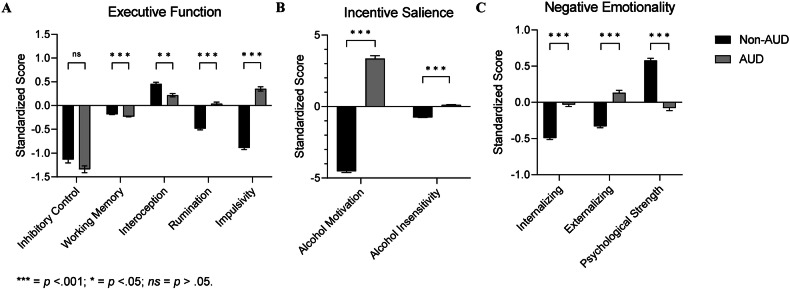
Differences in neurofunctional domain factors between individuals with and without AUD. Standardized factor scores from the (**A**) Executive Function, (**B**) Incentive Salience, and (**C**) Negative Emotionality domain factors, adjusted for age, sex, and race, between individuals with and without AUD.

**Fig. 4:**
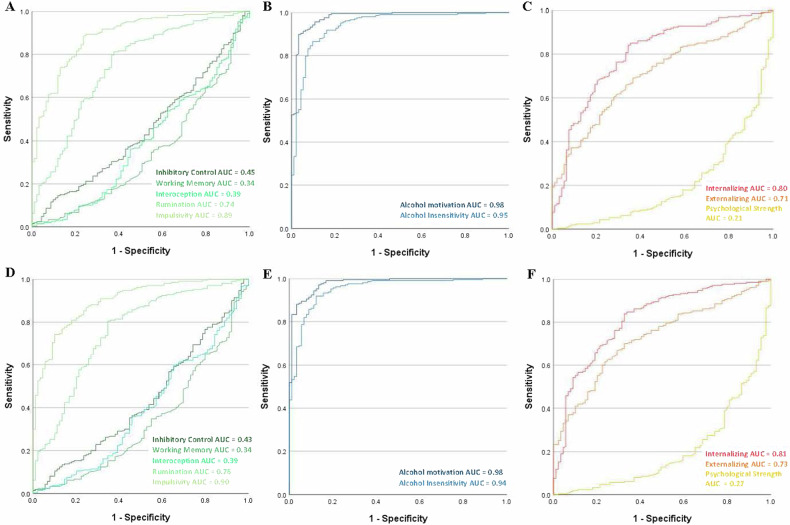
Classification ability of the neurofunctional domain factors. ROC curves for each domain factor predicting problematic drinking (**A**–**C**) and current AUD status (**D**–**F**). Problematic drinking = AUDIT score >8.0. AUD alcohol use disorder, ROC receiver operating characteristic, AUC area under the curve.

Standardized factor scores extracted from the full model exhibited distinct heterogeneity across individuals and diagnostic groups (Fig. 2). Furthermore, significant differences between AUD diagnosis groups were detected for all factors (all *p*’s < 0.002) except for inhibitory control (*p* = 0.19) (Fig. 3). Finally, ROC analysis revealed that both IS factors, alcohol motivation and alcohol insensitivity, showed excellent ability to discriminate individuals with problematic drinking (AUC = 0.98 and 0.94, respectively) and individuals with AUD (AUC = 0.98 and 0.95, respectively) (Fig. 4). For the other two domains, impulsivity (EF) and internalizing (NE) had the strongest ability to discriminate problematic drinkers (AUC = 0.89 and 0.80 respectively) and individuals with AUD (AUC = 0.90 and 0.81 respectively).

## Discussion

In the present study, an assessment battery specifically devised to assess the three ANA neurofunctional domains of incentive salience, negative emotionality, and executive function, was tested in a sample of adults representing diverse drinking behavior across the AUD spectrum. Unique factors underlying each of the ANA domains, and previously independently associated with AUD including alcohol insensitivity [21] and impulsivity [30] were identified and were shown to exhibit considerable variation both between individuals and between AUD diagnostic groups. This variability, and the ability to integrate previously identified determinants of AUD into ANA, underscores the clinical utility of the ANA framework to identify and better understand the heterogeneity of AUD.

Two IS domain factors were identified. Alcohol motivation encompassed measures of compulsion, craving, and a persistence to consume alcohol across varying costs (demand elasticity). Of note, anxiety and depression were also indicators of alcohol motivation, consistent with prior work [12] and in accordance with the self-medication hypothesis where alcohol consumption is driven by the need to relieve dysphoria [31]. Alcohol insensitivity captures an individual’s response to the effects of alcohol and amount of consumption when there is zero cost (demand intensity), and the findings here are consistent with data showing individuals with a low response to alcohol require more drinks to achieve their desired effect, which can lead to problematic alcohol use [21]. Three NE domain factors were identified: internalizing, externalizing, and psychological strength. Internalizing captured negative affect, anxiety, depression, and neuroticism, similar to previous works validating the NE domain [13, 16–18]. Externalizing captured aggression measures and low agreeableness. Chronic consumption of alcohol is linked to emotion dysregulation and behavioral disinhibition, which may increase the likelihood of engaging in aggressive behaviors after alcohol use [32, 33]. Finally, psychological strength captured the ability to adapt to stress and adversity, reflected by greater resilience and positive emotionality. Individuals high in psychological strength generally show higher coping abilities in the face of stressful events, which can buffer them against the development of psychopathology including SUDs [34].

The EF domain included five factors: inhibitory control, working memory, interoception, rumination, and impulsivity. Notably, inhibitory control and working memory primarily captured performance from the behavioral tasks while rumination, interoception, and impulsivity were measured using self-report scales. Previous studies have similarly identified multiple factors underlying executive functioning tasks, for example updating (monitoring and updating of working memory contents), shifting (switching between tasks/mental sets), and inhibition (conscious inhibition of dominant/prepotent responses) [20]. In the current study, the working memory factor is concordant with updating and shifting, while the inhibitory control factor is consistent with inhibition. Rumination, defined as negative, persistent, repetitive thoughts, is a maladaptive response to negative emotions [35], and has been linked to problematic alcohol use [36, 37]. Interoception refers to an awareness and integration of internal and external stimuli to regulate behavior, and modulates the likelihood of individuals approaching drug stimuli [38]. Lastly, impulsivity is a well-recognized multifaceted construct implicated in all stages of SUDs [30]. Taken together, these results highlight the multifaceted nature of executive functioning, extending it beyond traditional theories (i.e. [20]) and providing additional support for the role of executive dysfunction in the etiology of AUD.

Varying degrees of association were observed among the identified domain factors. Alcohol motivation, internalizing, rumination and impulsivity showed the strongest associations across factors, suggesting these might underlie core processes underlying the transition to AUD. Internalizing’s strong associations with rumination and impulsivity reflects an important link between the NE and EF domains. Individuals may respond to negative emotions by ruminating, which exacerbates negative emotionality [39]. Internalizing is also a component of negative urgency, a facet of impulsivity that is hypothesized to drive the transition to compulsive drug use [40]. Impulsivity was associated with most of the factors and, via its strong relationships with both alcohol motivation and internalizing, embodies a key link between all three domains. Alcohol motivation, alcohol insensitivity, and impulsivity also showed exceptional ability to identify individuals with problematic alcohol use and AUD. This highlights the key role impulsivity may play in the etiology of AUD and how impulsivity, together with alcohol motivation and internalizing, may serve as key endophenotypes for each of the stages of addiction [1, 11]. On the other hand, psychological strength’s positive association with interoception, and negative associations with rumination, alcohol motivation, and internalizing, highlight the importance of resiliency. Altogether, these findings suggest that negative emotionality can manifest itself differently across individuals (inwards/internalizing vs. outwards/externalizing), and that interventions targeted towards building psychological strength could potentially lessen the impact of negative emotionality on problematic drinking.

Individuals with AUD showed higher levels of alcohol motivation, alcohol insensitivity, internalizing, externalizing, rumination, and impulsivity, and lower levels of psychological strength, working memory, and interoception relative to those without AUD. Inhibitory control, as captured by the Stop Signal Task (SST), did not show differences between AUD groups. Previous studies looking at differences in SST performance among problematic drinkers have produced mixed findings [41, 42]. In an fMRI study comparing individuals with AUD and matched controls, Li et al. found that although the two groups did not differ in stop signal reaction time, those with AUD exhibited greater activity in their visual and frontal cortices [41]. This implies that individuals with AUD may exhibit altered neural processing compared to individuals without AUD in the absence of behavioral differences in inhibitory control.

In the present study, behavioral tasks tended to show poor correlations with self-report measures, even though they purportedly assess the same construct (e.g., self-reported impulsivity and delay discounting rate *k*). As such, variables derived from behavioral tasks either formed factors with each other, such as Inhibitory Control and Working Memory, or were not retained during the factor analysis. There are several possible sources of this discrepancy [43]. Behavioral tasks, which were constructed to minimize between-subject variability, generally exhibit poor reliability relative to self-report assessments. Poor reliability yields poor correlation. Additionally, behavioral tasks and questionnaires recruit distinct response processes; while behavioral tasks tap into actual performance, self-report questionnaires assess an individual’s perception of performance. Finally, questionnaires measure an individual’s assessment of their typical behavior, while behavioral tasks captures performance that reflects motivation in addition to ability [43]. However, these issues do not discount the utility of behavioral tasks in the ANA battery. While some of the behavioral tasks were ultimately not retained in the present analysis, their validity has been established from prior studies [42, 44–51]. Additionally, these instruments can be employed in functional neuroimaging studies to understand the underlying neurobiology of the ANA domains. Finally, while poor between-subject variability limits its use in detecting individual differences, these instruments may prove useful in assessing within-subject changes, such as during treatment recovery.

The present work extended the ANA framework in several ways. First, we replicated the three intercorrelated domain factors found by Kwako et al. [12] while further decomposing them into underlying constructs. We then identified three underlying factors that likely drive the correlations between domains: alcohol motivation (IS), internalizing (NE), and impulsivity (EF). The use of a comprehensive assessment battery also elucidated additional factors pertinent to AUD etiology (e.g., psychological strength). Second, the inclusion of behavioral tasks in the assessment battery provides opportunities for the ANA framework to be incorporated into various study designs. For example, functional neuroimaging studies can utilize these assessment tools to probe the neurobiological underpinnings of the domains. The basal ganglia, amygdala, and prefrontal cortex circuits are potential neurobiological correlates of the IS, NE, and EF domain factors respectively [1], and evaluating functional connectivity between these areas could provide important insights into the cycle of addiction.

Importantly, this work provides a critical step towards the primary objective of the ANA framework: to address heterogeneity within AUD and improve addiction phenotyping. The identified factors showed differing levels of individual variability (Fig. 2), suggesting that specific factors within each domain may be more pertinent in identifying subgroups. While the present study is underpowered to identify these subgroups, future studies can use person-centered approaches, such as latent profile analysis, to cluster individuals [52]. Additionally, future work would also be needed to test the predictive validity of these identified factors. If specific factors are associated with greater risk of relapse, the ANA battery can be used to identify the most vulnerable individuals. Identifying distinct subgroups and the key areas of dysfunctions allows clinicians to target these specific dysregulated processes, an important step towards precision medicine and improving treatment outcomes.

The present study has both strengths and limitations. Strengths include the range of neurocognitive behavioral tasks and clinical assessments that were specifically devised to elucidate the multidimensional structure of the ANA domain, the good psychometric properties attributed to these assessments, and a study sample comprising individuals with diverse drinking behavior across the AUD spectrum. Limitations include the cross-sectional design and the weak correlations between the self-report measures and behavioral tasks that purportedly measure the same construct (e.g., impulsivity). The cross-sectional design precludes determining whether these factors are antecedents or consequences of AUD. The extent to which dysfunctions in ANA domains preexist or are consequent to AUD and part of the process of addiction are likely to vary between individuals with AUD. The traits, such as alcohol sensitivity, impulsivity, internalizing behavior (negative emotionality) and executive cognition that indicate ANA domains are moderately to highly heritable, and are increasingly predicted by polygenic scores (PGS) derived from genome wide association studies, as is AUD itself [53]. Thus, these traits can be representative of domains of preexisting vulnerability. Yet, each of these traits is enduringly and often strongly altered by addiction, and only partly reversed during abstinence and recovery [1] thus representing a continuing basis of vulnerability to lapse and relapse. Future directions can include integration of PGS measures of liability and exposure both retrospectively, and in longitudinal, prospective studies (e.g., National Consortium on Alcohol & Neurodevelopment in Adolescence or NCANDA [54], Adolescent Brain Cognitive Development or ABCD study [55]). Finally, the weak correlations between self-report measures and behavioral tasks have previously been recognized, and are due to behavioral measures having relatively low reliability and not sharing the same response process as self-report measures [43]. Regardless, the identification of neurocognitive assessments that were differentiated by AUD status can inform subsequent neuroimaging research and ecological momentary assessment studies. Whether the identified ANA factors generalize to other SUDs remains unknown. While IS measures in the present study were alcohol-specific, the NE and EF factors are implicated in other SUDs. Future studies are needed to assess the specificity of these identified factors.

In conclusion, this primary investigation evaluated a standardized battery of neurocognitive assessments designed to assess the three neurofunctional domains of ANA and identified multiple inter-correlated factors underlying each domain, including factors such as alcohol insensitivity and impulsivity that independently predict AUD. These factors, as well as their relationship with each other, differed between individuals with and without AUD and may reflect core underlying processes that lead to or maintain AUD. With an average completion time of 2–3 h and a 98% completion rate, we found preliminary evidence supporting the feasibility of administering this standardized ANA battery in a clinical research setting. Future work is needed to develop a shorter assessment battery to improve clinical utility, identify relevant neurobiological correlates, and uncover subgroups of individuals with AUD based on these factors using person-centered approaches (e.g., latent profile analysis) to better characterize diagnostic heterogeneity and inform precision medicine.

### Supplementary information

Supplemental Tables

## Data Availability

The data that support the findings of this study are available upon reasonable request from the corresponding author.
